# Genomic fingerprints of *Escherichia coli* strains isolated from surface water in Alborz province, Iran

**DOI:** 10.1186/s13104-017-2575-z

**Published:** 2017-07-20

**Authors:** Reza Ranjbar, Parichehr Pezeshknejad, Faham Khamesipour, Kiumars Amini, Roohollah Kheiri

**Affiliations:** 1 Molecular Biology Research Center, Baqiyatallah University of Medical Sciences, Tehran, Iran; 2 Department of Microbiology, Saveh Science and Research Branch, Islamic Azad University, Saveh, Iran; 3 Cellular and Molecular Research Center, Sabzevar University of Medical Sciences, Sabzevar, Iran; 4 Health Policy Research Center, Institute of Health, Shiraz University of Medical Sciences, Shiraz, Iran; 5 Water Quality Control Office, Alborz Province Water and Wastewater Company, Karaj, Iran

**Keywords:** *Escherichia coli*, Fingerprinting, Surface water

## Abstract

**Background:**

Consistent use of suitable diagnostic methods is essential to evaluate the genomic diversity of *E. coli* strains. Advance of efficient methods to discriminate the causes of *E. coli* in aquatic environments is important. This study aimed to describe the strain diversity of an *E. coli* population retrieved from surface water.

**Methods:**

One hundred water samples were drawn within a period of 1 year, from May 2012 to May 2013, and *E. coli* bacteria have been isolated from water samples. The genomic diversity analysis of 100 isolates of *E. coli* (one isolate per sample) has been carried out with the use of the ERIC-PCR fingerprinting method.

**Results:**

Overall, our data indicated that complex fingerprint patterns have been obtained for totally of the isolates. Highest number of strains were in E4 (20 strains with more than 20% similarity) and lowest number of strains were in E3 (5 strains) group. In addition, there was no similarity in E1 (9 strains), E8 (10 strains) and E9 (7 strains) clusters.

**Conclusion:**

Therefore, the occurrence of potential pathogenic *E. coli* and diversity of *E. coli* strains in surface water in Alborz province, Iran could pose a possible risk to animal health and human if not disinfected well.

## Background

*Escherichia coli* (*E. coli*) is a consistent dweller of the human intestinal tract, and it is the chief facultative organism in the human gastrointestinal tract [1–4]. One device for confirming being in the environment might be a variance biofilm-forming skill in some natural *E. coli* inhabitants. Though biofilm formation is the remaining result of multiple interacting molecular events [5–8] and is greatest usefully measured at the phenotypic level, a lack of detached genetic systems may be significant to adhesion properties, and some inhabitants level variability in their occurrence performs routine of study.

At the genotypic level, there are two phase-variable surface proteins, antigen 43 (Ag43) and type 1 fimbriae, encoded by the *fim* gene cluster [9, 10] and Ag43 encoding gene (agn 43) [11], respectively, which have been recommended as thoughtful in determining the adhesion properties of *E. coli*.

A number of high-resolution molecular fingerprinting methods have been used to reveal species and subspecies diversity [12–14]. PCR-based marker methods have been employed lengthily for approving genotypes of organisms at the level of species and populace. The PCR technique needs slight biological material and make available a quick technique for showing large sample sizes. PCR markers have been developed by moreover random primers or specifically designed primers from identified DNA info for instance repetitive [15].

Genomes of eukaryotes cover a large amount (50–80%) of repeated DNA sequences, simple sequence repeats (SSRs) referred to mini satellite DNA and interspersed repeated sequences together with Alu and inter simple sequence repeats (ISSRs) [16–18]. Moreover, repetitive portions for instance enterobacterial repetitive intergenic consensus (ERIC) [19–21] and repetitive extragenic palindromic (REP) [22] are as well established in prokaryotic genomes. These repeated DNA have been utilized to generate PCR primers valuable in detecting genetic variations in animals, plants, and microbes amid or inside species [23–25] ever since of the highly variable nature of the loci.

Advance of efficient methods to differentiate the sources of *E. coli* in aquatic environments is important to recover the observation of fecal contamination indicators, to improve plans to detect the sources of fecal infection, and to device suitable running performs to decrease gastrointestinal disease transmission [26].

Various DNA fingerprinting techniques exists among which, repetitive extra-genic palindromic elements-polymerase chain reaction (REP-PCR) make available great taxonomic resolution and can act as a fast detector of diversity and progress of the microbial genomes [27]. Among REP fingerprinting methods (REP/ERIC/BOX), ERIC PCR is more preferred because of simple protocol and discriminatory power similar to PFGE [28]. So, the aims of the current research were to describe the strain diversity of an *E. coli* populace recovered from surface water via employing a whole-genome fingerprinting method.

## Methods

### *E. coli* source and water sampling

Alborz province with 2.413 million populations is located in south west of Tehran province in central region of Iran. Amirkabir dike and Karaj River are the main sources of water in this province.

This descriptive cross-sectional study was made from May 2012 to May 2013. The authors collected 100 water samples from a given sampling site in Karaj River (with geographic coordinate of 35.9404423, 51.0742861) according to standard microbiological sampling protocols (APHA 2012). They were directly placed in a lightproof insulated box inclosing ice-packs to make sure fast cooling and shipped to the laboratory. The determination of the fecal coliform has been carried out with the use of 9221-A and 9221-D. To isolate *E. coli*, water samples were inoculated into lauryl tryptose broth (Merck KGaA) followed by *E. coli* (EC) broth (Merck KGaA) at 44.5 ^°^C and streaked onto eosin methylene blue (EMB) agar (Merck KGaA) [29]. Colonies display metal sheen were considered as presumptive *E. coli* isolates and were subjected to IMViC (Merck KGaA), glucuronidase, and tryptophanase (Merck KGaA) tests for last approval [29]. All recovered *E. coli* isolates were put in storage at −70 °C in brain heart infusion (BHI) broth comprising 15% glycerol for further use.

### DNA extraction and ERIC PCR

We had evaluated one hundred isolates of *E. coli* which were separated from Alborz province water sources. Genomic bacterial DNA of 95 *E. Coli* isolates was extracted via column based technique as described previously by Tolosa (2007) [30]. ERIC-PCR was performed with the two primer sequences of ERIC1 (5′-ATGTAAGCTCCTGGGGATTCAC-3′) and ERIC2 (5′-AAGTAAGTGACTGGGGTGAGCG-3′) as described previously [31]. The 25 µl PCR mixture included 17.5 µl distilled water, 2.5 µl 10× PCR buffer, 1.25 µl MgCl_2_ (2.5 mM), 0.5 µl dNTP (200 µM), 1 µl from each primer ERIC1 and ERIC2, 0.4 µM), 0.25 µl Taq DNA polymerase (1.25 U) and 1 µl template DNA. PCR amplification was performed using an Eppendorf Thermal Cycler as follows: initial denaturation at 94 °C for 5 min, 30 cycles of denaturation at 94 °C for 30 s, annealing at 52 °C for 40 s, and extension at 72 °C for 5 min followed by a last extension at 72 °C for 5 min. Then, electrophoresis was performed using 1.5% agarose gel, at 70 v for 2 h and dyed with ethidium bromide. Credibility of the ERIC-PCR patterns for each *E. coli* isolate was proved using parallel runs on separate occasions but on the same thermo cycler. Finally, the loaded gels were visualized by Gel DOC™ XR^+^ (BIORAD), banding patterns and size were determined via Image Lab ™ 4.0.

## Results

**Fig. 1:**
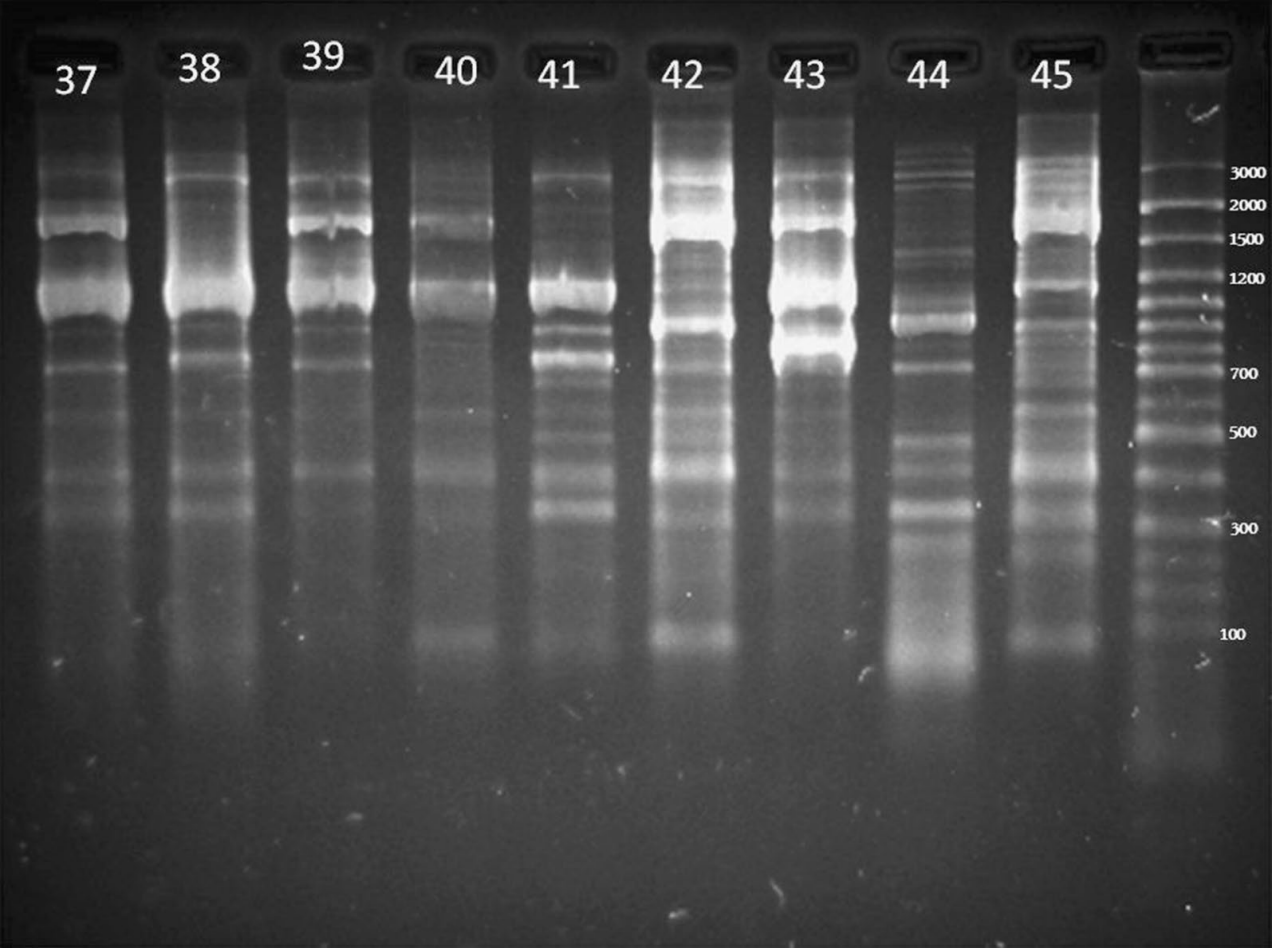
ERIC-PCR fingerprinting patterns of the isolates. *Lane MW* GeneRuler 100 bp plus DNA Ladder (Thermo Scientific). *Lane 37*–*45* ERIC fingerprints of *E. coli* isolates

**Fig. 2:**
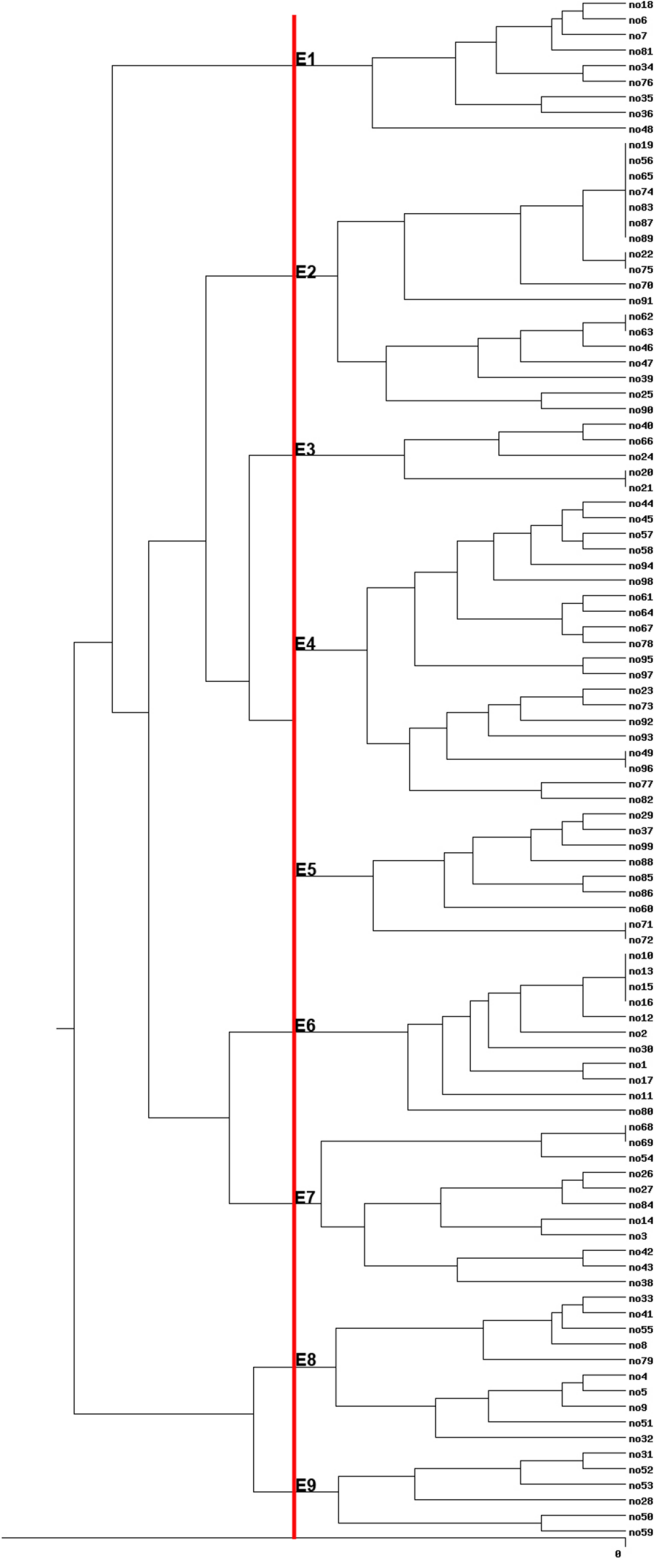
Cluster analysis by ERIC-PCR fingerprint of 100 *E. coli* isolates. The *bottom bar* indicates the isolates number, while the *right bar* indicates the percentage of similarity. Cluster analysis at a coefficient of 75% similarity (*red line*), grouped 100 isolates into 9 clusters, designated E1–E9. Each cluster has demonstrated a characteristic number of strains as well as separate inter- and intra-group similarity relations

One hundred *E. coli* were isolated from 100 water sample indicating high fecal pollution of river. The genomic diversity analysis of 100 isolates of *E. coli* has been carried out with the use of the ERIC-PCR fingerprinting method. Complex fingerprint patterns have been found for totally of the isolates studied. A typical fingerprint for some *E. coli* isolates generated by ERIC PCR has been shown in Fig. 1. Then, our data were inserted in http://insilico.ehu.es/PCR site to construction of the dendrogram. The received dendrogram has grouped the 100 strains of *E. coli* into nine similarity groups with 75% similarity (Fig. 2).

The percentages of *E. coli* strains, according in dendrogram, are summarized in Table 1. Highest number of strains were in E4 (20 strains with more than 20% similarity) and lowest number of strains were in E3 (5 strains) group. 18 strains with 75% similarity were belonging to E2 group. In addition, there was no similarity in E1 (9 strains), E8 (10 strains) and E9 (7 strains) between their strains.

**Table 1:** The percentage of *E. coli* strains, according in dendrogram

Group	E1	E2	E3	E4	E5	E6	E7	E8	E9
Number	9	18	5	20	9	11	11	10	*7*
Percent	9	18	5	20	9	11	11	10	*7*

## Discussion

One hundred *E. coli* clones isolated from surface water were involved in the genomic diversity analysis. The detection of *E. coli* in water is an implicit indicator of fresh fecal pollution and consequently of the hazard of co-occurrence of enteric pathogens that can reason illness in susceptible populations [32] and the great ERIC diversity obtained in this study confirms the entirely different sources of water contamination, because river as a non-selective media can receive pollution from many sources.

In the current study, complex fingerprint patterns have been obtained for all *E. coli* isolates using ERIC PCR. Moreover, the received dendrogram has grouped the 100 strains of *E. coli* into nine similarity groups with 75% similarity. Our finding is in line with a study conducted to analyse the genomic diversity in *E. coli* strains isolated from surface water using rep-PCR fingerprinting technique, by the support of REP and ERIC primers. The result showed that, the great genomic diversity of the *E. coli* of the surface region was expressed as a dendrogram in the form of eight similarity groups including strains isolated from samples drawn above 1 month. The bottom-zone strains, which show a lesser degree of genomic diversity (5 similarity groups), presented different communal structures in their DNA fingerprints. In the similarity dendrogram for the bottom-zone, strains resulting in changed months of sampling were separated hooked on the similar similarity groups [33].

The percentages of *E. coli* strains, according in dendrogram, in our study showed highest number of strains were in E4 (20 strains with more than 20% similarity) and lowest number of strains were in E3 (5 strains) group. Eighteen strains with 75% similarity were belonging to E2 group. In addition, there was no similarity in E1 (9 strains), E8 (10 strains) and E9 (7 strains) between their strains. Another study conducted on the relative usefulness of five different rep-PCR techniques to discriminate *E. coli* inhabitants indicated that, cluster analysis of ERIC-PCR and ERIC2-PCR profiles of 270 *E. coli* revealed 23 clusters and 14 clusters, respectively. The discriminant analysis of rep-PCR genomic fingerprints of 271 *E. coli* isolates yielded an regular rate of correct classification (watershed-specific) of 72.6 and 55.8% for ERIC-PCR and ERIC2-PCR, respectively [26]. So, the genomic diversity in *E. coli* strains put on REP primers in rep-PCR generates extra complex fingerprints increasing the discriminatory power of the analysis, whereas the ERIC primer generates less complex fingerprint patterns, and is thus clearer to interpret.

## Conclusion

Accessory gene fingerprinting may have significant applied suggestions for improving the specificity of techniques that are usually used to describe the strain diversity of an *E. coli* population from surface water. ERIC-PCR is an appropriate method to rapidly evaluate the genetic diversity of *E. coli* strains. To, by this assay, genomic fingerprints from *E. coli* isolates were distinct and displayed differences in the number of bands, intensity and fragment size.

## Abbreviations

agn 43: ag43 encoding gene
BHI: brain heart infusion
ERIC: enterobacterial repetitive intergenic consensus
*E. coli*: *Escherichia coli*
ISSRs: Alu and inter simple sequence repeats
PCR: polymerase chain reaction
REP: repetitive extragenic palindromic
